# Longitudinal trends in enrollees’ employment and student status after Medicaid expansion

**DOI:** 10.1186/s12913-022-07599-x

**Published:** 2022-02-19

**Authors:** Renuka Tipirneni, Edith C. Kieffer, John Z. Ayanian, Minal R. Patel, Matthias A. Kirch, Jamie E. Luster, Monita Karmakar, Susan D. Goold

**Affiliations:** 1 University of Michigan Institute for Healthcare Policy and Innovation, Ann Arbor, MI 48109 USA; 2 Department of Internal Medicine, University of Michigan, 2800 Plymouth Road, Bldg 16, Rm 419W, Ann Arbor, MI 48109 USA; 3 School of Social Work, University of Michigan, Ann Arbor, MI 48109 USA; 4 School of Public Health, University of Michigan, Ann Arbor, MI 48109 USA; 5 University of Michigan Gerald R. Ford School of Public Policy, Ann Arbor, MI 48109 USA; 6 Center for Bioethics and Social Sciences in Medicine, University of Michigan, Ann Arbor, MI 48109 USA

**Keywords:** Medicaid, Employment, Health reform, Health policy

## Abstract

**Background:**

Medicaid community engagement requirements previously received federal approval in 12 states, despite limited data on their impact on enrollees’ employment-related activities. Our objective was to assess longitudinal changes in enrollees’ employment and student status after implementation of Michigan’s Medicaid expansion.

**Methods:**

Longitudinal telephone survey of Michigan Medicaid expansion enrollees in 2016 (response rate [RR] = 53.7%), 2017 (RR = 83.4%), and 2018 (*N* = 2,608, RR = 89.4%) serially assessing self-reported employment or student status. Survey responses were benchmarked against statewide changes in assessed similar low-income adults in the U.S. Census Bureau Current Population Survey. We used mixed models with individual random effects to assess changes in the proportion of enrollees who were employed or students by year.

**Results:**

Most respondents had incomes < 100% FPL (61.7% with 0–35% of the federal poverty level [FPL], 22.9% with 36–99% FPL, and 15.4% with 100–133% FPL), 89.3% had at least a high school diploma/equivalent, and they ranged in age (39.6% age 19–34, 34.5% age 35–50, 25.9% age 51–64). Employment or student status increased significantly among Michigan Medicaid expansion respondents, from 54.5% in 2016 to 61.4% in 2018 (*P* < 0.001), including among those with a chronic condition (47.8% to 53.8%, *P* < 0.001) or mental health/substance use disorder (48.5% to 56.0%, *P* < 0.001). In contrast, the statewide proportion of low-income non-elderly adults who were employed or students did not change significantly (from 42.7% in 2016 to 46.0% in 2018, *P* = 0.57).

**Conclusions:**

Medicaid expansion, absent a community engagement requirement, was associated with increased employment and related activities. The role of Medicaid in providing safety-net coverage to individuals during times of economic stress is likely to grow.

## Introduction

In 2018, the U.S. Centers for Medicare and Medicaid Services (CMS) wrote to state Medicaid directors encouraging Sect. 1115 waiver applications to test incentives requiring work or other community engagement for continued Medicaid eligibility for certain adult Medicaid beneficiaries. [1] The premise within the CMS memo was that increased community engagement resulting from these requirements would lead to improved physical and mental health, emergence from poverty, and increased financial independence among Medicaid beneficiaries [1]. CMS wrote that these anticipated outcomes were in line with the objectives of the Medicaid program.

Subsequently, Medicaid community engagement requirements (participation in work, school, job training, job searching, or volunteering) received federal approval in twelve states. However, a federal court halted work requirement policies in Michigan and other states, and in 2021 the Biden administration began the process of withdrawing these waiver authorities. Nonetheless, data documenting trends in Medicaid enrollees’ engagement in work-related activities are limited. Our objective was to assess longitudinal changes in enrollees’ employment and student status after implementation of Michigan’s Medicaid expansion. (the “Healthy Michigan Plan” [HMP]).

## Methods

Institutional Review Boards deemed this public program evaluation exempt from review.

### Data Sources

#### Healthy Michigan Voices enrollee survey

We conducted a telephone survey of a longitudinal panel of Medicaid expansion enrollees, ages 19–64 with incomes up to 133% of the federal poverty level (FPL), effectively up to 138% FPL after application of the Medicaid 5% income disregard. Details of the Healthy Michigan Voices Survey, including sampling and design, have been described in previously published work [2–4]. The 2016 survey response rate was 53.7% (*N* = 4,090) and follow-up response rates among those who agreed to be recontacted were 83.4% in 2017 (*N* = 3,104) and 89.4% in 2018 (*N* = 2,608), with an overall response rate of 39.5% across the three survey waves [5]. The present study population included in analysis was only individuals who responded to all three waves of the survey in 2016, 2017 and 2018. While all individuals sampled in the 2016 survey were currently enrolled in HMP, individuals remained in our survey cohort whether or not they were enrolled in HMP in follow-up years, 2017 and 2018; thus, all 2017 and 2018 survey estimates reflect a combination of current and previous enrollees in the program.

#### Census data

To examine whether observed trends among HMP respondents reflected changes attributable to the program or statewide secular trends, we benchmarked HMP enrollee survey data against data from low-income adults ages 19–64 in Michigan overall. Data were obtained from serial cross-sectional surveys in the U.S. Census Bureau Current Population Survey (CPS) Annual Social and Economic Supplement from 2016, 2017 and 2018. Low income was defined as persons with household income up to 138% FPL, which approximates HMP income eligibility of 133% FPL after applying the Medicaid 5% income disregard. We also conducted sensitivity analyses using the U.S. Census Bureau’s American Community Survey (ACS), using the same definitions as noted for the main CPS analyses.

### Measures

#### Enrollee survey data

Survey items measured employment (“Are you currently employed or self-employed?”, with response options yes/no) and student status (“Are you currently in school?”, with response options yes/no). For those not currently employed or in school full-time, a follow-up question asked, “Would you say you are: retired, unable to work, or out of work?”. Employed enrollees were asked their working hours (“Including overtime, about how many hours per week do you usually work on all paid jobs?”), while enrollees who were not employed were asked about job searching (“Are you currently looking for a job?”) or job training (“In the last 2 years, have you completed or are you currently enrolled in training, programs, or courses to increase your chances of getting or keeping a job?”). Respondents who reported inability to work were asked about reasons for being unable to work (“What are the reasons you are unable to work?”).

Health status was assessed in the enrollee survey by the items, “In general, would you say your health is…?” (with response options: excellent, very good, good, fair, or poor), “For how many days during the past 30 days was your physical health not good?”, “For how many days during the past 30 days was your mental health not good?”, and “During the past 30 days, for how many days did poor physical or mental health keep you from doing your usual activities, such as self-care, work, or recreation?”. Enrollee demographic characteristics were obtained from Medicaid enrollment files and survey items. Chronic conditions and mental health/substance use disorders were identified from survey and claims diagnosis data.

#### CPS data

Individuals who were civilians and reported any of the following were considered employed: work as paid employees, self-employed business/farm, worked at least 15 h as unpaid worker at business/farm owned by a family member, or had a job but were not currently working because of illness, weather, vacation, time off, or labor-management disputes. Individuals who reported they were enrolled in school full-time or part-time and had attended within the last week were considered students.

### Data analysis

For HMP survey analyses, we used mixed models with individual random effects to assess changes in the proportion of HMP respondents who were employed or students by year, incorporating weight adjustments for sample design, initial nonresponse, and post-stratification adjustment for non-response to the follow-up. The mixed effects regression allowed us to examine longitudinal changes in the same sample of HMP respondents across years. For the benchmarking analyses of low-income Michigan adults ages 19–64 in the CPS data, we used survey-weighted logistic regression to assess changes in the proportion of Michigan residents who were employed or students by year.

The mixed effects regression model assumes a linear relationship between the natural log of the probability of the outcome (employed or student) and the predictor variable (year) and no outliers. With only three years of data we cannot definitively demonstrate a linear relationship, but there were no outliers. The mixed effects model was deemed appropriate to assess change over time in the repeated measures of employment or student status for each individual person, as we assume employment or student status in one time may be linked to employment or student status at another time.

## Results

 Most HMP respondents had incomes < 100% FPL (61.7% with 0–35% FPL, 22.9% with 36–99% FPL, and 15.4% with 100–133% FPL), 89.3% had at least a high school diploma/equivalent; and they ranged in age (39.6% age 19–34, 34.5% age 35–50, 25.9% age 51–64) (Table 1). 36.7% of respondents reported that their health was excellent or very good, 37.5% good, 19.7% fair, and 6.1% poor (Table 1). The mean number of days of poor physical health in the last 30 days was 5.9 and the mean number of poor mental health days was 5.7. More than 60% of respondents had a diagnosis of a chronic condition (61.7%) or a mental health/substance use disorder (63.4%) (Table 1) .

**Table 1:** Demographic Characteristics of 2018 Healthy Michigan Plan Enrollee Follow-Up Survey Respondents

Characteristic	Weighted Proportion or Mean [95% CI] (*N* = 2,608)
Age^*^	
19–34	39.6 [37.2, 42.1]
35–50	34.5 [32.2, 36.8]
51–64	25.9 [24.1, 27.8]
Sex^*^	
Female	51.5 [49.1, 53.9]
Race/ethnicity^†^	
White, non-Hispanic	58.7 [56.4, 60.9]
Black, non-Hispanic	26.9 [24.8, 29.0]
Hispanic	4.5 [3.6, 5.6]
Other, non-Hispanic	9.9 [8.4, 11.6]
Income (% Federal Poverty Level)^*^	
0–35%	61.7 [59.6, 63.8]
36–99%	22.9 [21.1, 24.9]
100–133%	15.4 [13.8, 17.0]
Highest level of education^‡^	
Less than high school	10.7 [9.4, 12.3]
High school graduate (or equivalent)	39.8 [37.5, 42.1]
Some college	23.2 [21.2, 25.3]
Associate’s degree	13.1 [11.6, 14.8]
Bachelor’s degree	10.5 [9.1, 12.0]
Post graduate degree	2.7 [2.1, 3.5]
Marital status^§^	
Married or partnered	26.0 [24.1, 28.0]
Health status^§^	
Excellent	9.9 [8.5, 11.5]
Very good	26.8 [24.8, 29.0]
Good	37.5 [35.2, 39.8]
Fair	19.7 [17.8, 21.6]
Poor	6.1 [5.1, 7.4]
Mean number of days physical health not good in past 30 days^§^	5.9 [5.4, 6.3]
Mean number of days mental health not good in past 30 days^§^	5.7 [5.3, 6.2]
Mean number of days poor physical or mental health limited usual activities in past 30 days^§^	4.7 [4.3, 5.2]
Chronic condition^†,‡,§,‖^	
Yes	61.7 [59.3, 64.0]
Mental health and/or substance use disorder^†,‡,§,‖^	
Yes	63.4 [61.1, 65.7]
Employment status, detailed^§^	
Full-time employment	27.3 [25.2, 29.5]
Part-time employment	29.5 [27.4, 31.6]
Out of work	15.7 [14.0, 17.7]
Unable to work	18.5 [16.7, 20.3]
Retired	4.8 [4.1, 5.7]
Don’t know	4.2 [3.3, 5.3]
In school^§^	
Yes	9.3 [7.8, 10.9]
Student status among those in school^§^	
Full-time	57.7 [48.8, 66.2]
Part-time	42.3 [33.8, 51.2]

Source, Authors’ analysis of data from the Healthy Michigan Plan (HMP) enrollee survey, 2016, 2017, 2018

Notes:^*^Variable from 2016 Medicaid enrollment data

^†^Variable from 2016 HMP enrollee survey

^‡^Variable from 2017 HMP enrollee survey

^§^Variable from 2018 HMP enrollee survey

^‖^Variable from Medicaid administrative claims diagnosis data

**Fig. 1:**
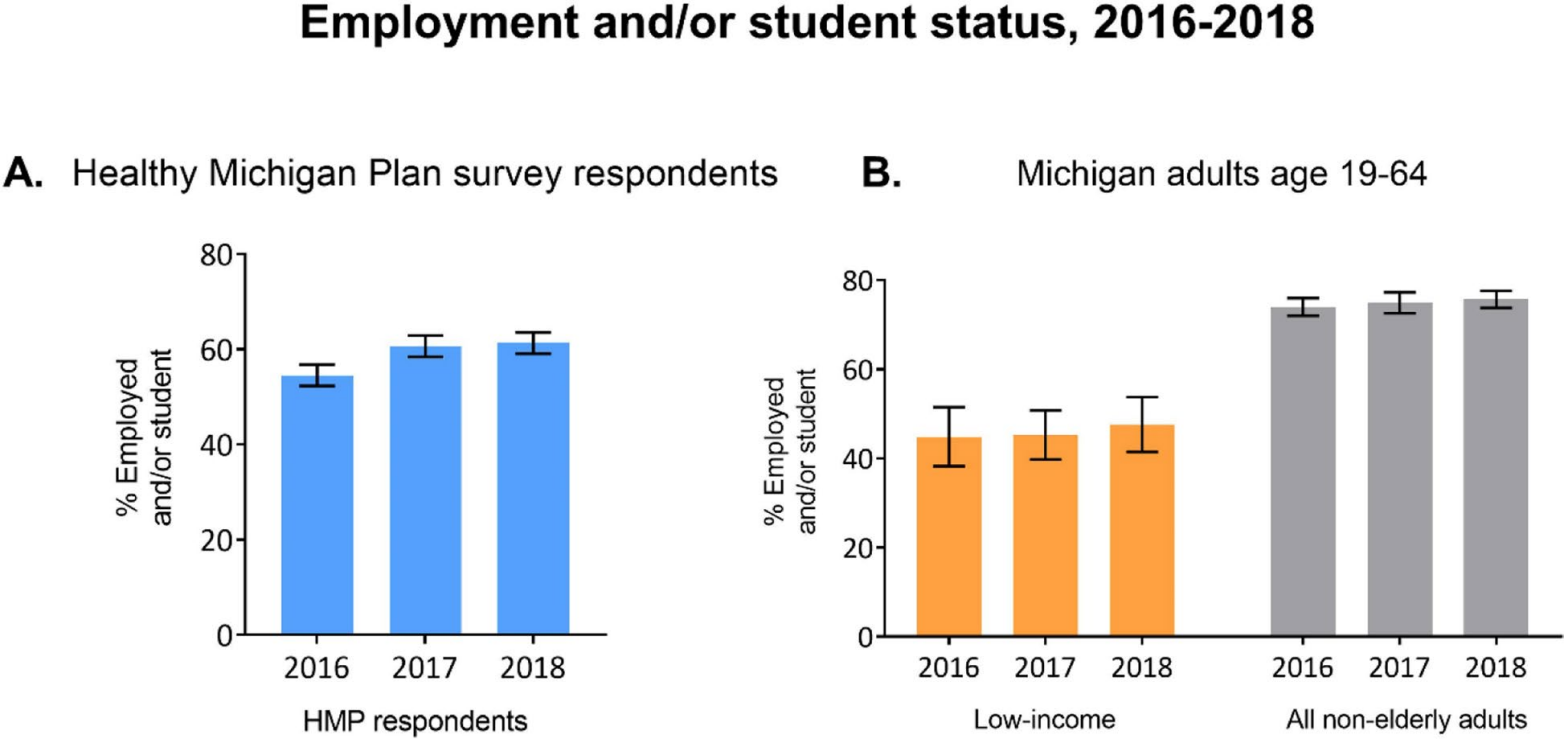
Longitudinal Trends in Employment and Student Status After Medicaid Expansion Among Michigan Medicaid Enrollees and Non-Elderly Michigan Adults. Source: Panel A, Authors’ analysis of data from the Healthy Michigan Plan (HMP) enrollee survey, 2016, 2017, 2018; Panel B, Authors’ analysis of data from the Current Population Survey Annual Social and Economic Supplement, 2016, 2017, and 2018 surveys. Notes: Panel A reports data from HMP survey respondents. Adults age 19-64 with incomes up to 133% of the federal poverty level (FPL) are eligible for HMP. Panel B reports data from low-income Michigan adults age 19-64 in the Current Population Survey. In Panel B, low income is defined as persons with household income up to 138% FPL, which approximates HMP income eligibility of 133% FPL after applying the Medicaid 5% income disregard. Weighted percentages are presented. Error bars indicate 95% CIs. Employed and/or student includes those who are employed full-time or part-time or are full-time or part-time students. *P* < 0.001 for employment or student status change from 2016 to 2018 for HMP survey respondents (Panel **A**), estimated from mixed effects logistic regression model with time indicators and no covariates. *P*=0.54 for Michigan low-income adults age 19-64 and *P*=0.22 for all non-elderly adults age 19-64 (Panel **B**), estimated from logistic regression models with time indicators and no covariates.

More than half of HMP respondents were employed (27.3% full-time and 29.5% part-time) and 9.3% were in school in 2018 (Table 1). From 2016 to 2018, the proportion of HMP respondents who were employed or students increased by 6.9 percentage points (54.5% to 61.4%, *P* < 0.001) (Fig. 1, Panel A), including among those with a chronic condition (47.8% to 53.8%, *P* < 0.001) and those with a mental health/substance use disorder (48.5% to 56.0%, *P* < 0.001). CPS data for Michigan showed that the proportion of low-income non-elderly adults who were employed or students did not change significantly over time (from 44.8% in 2016 to 47.5% in 2018, *P* = 0.54) (Fig. 1, Panel B). In sensitivity analyses, benchmarking against ACS data showed similar results to the CPS data.

Employed HMP respondents worked 34.7 mean hours per week on all paid jobs in 2018 (Table 2). 88.1% of employed HMP respondents, and 51.0% of all respondents, reported working 20 or more hours per week (Table 2). Among HMP respondents in school, 57.7% were full-time and 42.3% were part-time students (Table 1). HMP respondents who were not employed or full-time students in 2018 reported inability to work (47.3%), being out of work (40.4%) or retired (12.3%) (Table 2). HMP respondents who reported inability to work attributed their employment status to poor health/disability (91.4%) and caregiving responsibilities (7.1%) (Table 2). Among all HMP respondents who were not employed in 2018, 36.8% were searching for a job and 9.1% had completed or were enrolled in job training (Table 2).

**Table 2:** Activities of Employed and Non-Working Healthy Michigan Plan Enrollee Survey Respondents, 2018

	**Weighted Proportion or Mean [95% CI]**
Employed respondents (*N* = 1,471)	
Mean number of hours worked per week^*^	34.7 [33.8, 35.6]
Respondents who were not employed (*N* = 1,137)	
* Looking for a job*	36.8 [33.2, 40.7]
* Recently completed/enrolled in job training program*	9.1 [7.0, 11.8]
Respondents who were not employed or full-time students (*N* = 1,136)	
* Work status*	
Unable to work	47.3 [43.6, 51.1]
Out of work	40.4 [36.6, 44.2]
Retired	12.3 [10.5, 14.4]
Respondents who were unable to work (*N* = 526)	
*Reasons unable to work*^*†*^	
Poor health/disability	91.4 [87.8, 94.0]
Caregiving responsibilities	7.1 [4.8, 10.3]
In school or other training	1.1 [0.3, 3.9]
Prior conviction	0.6 [0.1, 4.3]
Transportation/logistics	0.5 [0.1, 2.0]
Couldn’t find work	0.5 [0.1, 2.0]
Other	0.2 [0.0, 0.7]
Employers think I’m too old or too young	0.2 [0.0, 1.2]

Source: Authors’ analysis of data from the Healthy Michigan Plan (HMP) enrollee survey, 2018

Notes: ^*^88.1% of employed HMP survey respondents, and 51.0% of all HMP survey respondents, reported working 20 or more hours per week

^†^Respondents were able to provide multiple responses

## Discussion

From 2016 to 2018, employment or student status increased significantly among Michigan Medicaid expansion enrollees, including those with comorbid conditions, while overall employment or student status for low-income adults in the state did not. This increase occurred prior to implementation of the state’s community engagement requirement in January 2020, which was later halted by a federal court in March 2020.

Prior studies of the potential effects of Medicaid expansion on employment have yielded mixed results. While several studies of specific states, including Michigan and Ohio, [2–4, 6] or specific sub-groups such as individuals with disabilities [7, 8] have shown evidence of positive employment-related outcomes, other robust national studies using federal data such as the CPS and ACS have overall found no significant effects on employment [9–13]. The differences in findings could be related to differences in study design. In addition, the national studies only used data through 2015 or 2016, so it is possible that data from more recent years could uncover longer-term effects of Medicaid expansion on employment.

A previous study of employment and Medicaid, using 2015–2016 national survey data with self-reported Medicaid status, found that three-quarters of non-elderly Medicaid beneficiaries were employed at some point during enrollment but a third of these were employed only some months of the year [14]. The authors suggested that many beneficiaries might have difficulty meeting monthly community engagement requirements. While many were employed when they left Medicaid, 21% did not gain employment after leaving. The report also found that almost a quarter reported health declines or loss of a job prior to Medicaid enrollment. Another recent study found that Medicaid enrollees not meeting work requirements were sicker than those meeting requirements, suggesting health-related barriers to work not completely accounted for by medical exemptions [15]. These studies demonstrate that many Medicaid enrollees may have inconsistent employment even if employed or students at some point in the year, which highlights the importance of Medicaid for maintenance of health and functioning, as well as the vulnerability to health shocks should enrollees fail to meet monthly community engagement requirements. At present, four of the approved state community engagement waivers have been set aside by a federal court — including Michigan’s — and the Biden administration plans to remove all other waiver authorities for community engagement requirements. In Arkansas, the only state to have fully implemented such a requirement, enrollees experienced no change in employment but significant drops in coverage 6 months after implementation [16].

### Limitations

Potential study limitations include, first, survey non-response bias though we applied survey weight and post-stratification adjustments for non-response. Second, our surveys included no assessment of employment or student status prior to Medicaid expansion. Third, minor changes in survey questions on employment or student status could contribute to observed changes in employment status, though we consider this less likely. Fourth, our follow-up survey cohort included some individuals who were no longer enrolled in HMP or another Medicaid program at the time of the survey, but this constituted a minority of the sample (less than 25%). Fifth, we were limited to sampling in a single state, although Michigan is one of the few states that had an approved community engagement waiver. Sixth, observational data without a comparison group limits causal inference.

### Policy implications

Our findings suggest that Medicaid expansion enrollees were more likely to be employed or students four years after implementation of the HMP program in 2014. The massive involuntary job losses across the country in the wake of the COVID-19 pandemic reveal the vulnerability of Medicaid beneficiaries subject to work requirements to loss of coverage. Even without a pandemic, economic downturns make seeking and maintaining employment challenging and sometimes unattainable. The nature of most low-wage employment is unstable, part-time, or with unpredictable hours. In addition, many low-wage jobs do not provide health insurance. Previous studies highlight the importance of Medicaid expansion for promotion of good health and functioning, which facilitate seeking and maintaining employment, [2–4, 17–19] though the national evidence remains mixed on the association with employment outcomes overall. While the policy of Medicaid community engagement requirements may no longer be implemented in the coming years, the role of Medicaid in providing safety-net coverage to individuals during times of economic stress is likely to grow.. 

## Data Availability

All data generated or analysed during this study are available in the following repository: https://deepblue.lib.umich.edu/handle/2027.42/155552
